# Follow-up of the manganese-exposed workers healthy cohort (MEWHC) and biobank management from 2011 to 2017 in China

**DOI:** 10.1186/s12889-018-5880-0

**Published:** 2018-08-01

**Authors:** Yanting Zhou, Xiaoting Ge, Yuefei Shen, Lian Qin, Yaoqiu Zhong, Chao Jiang, Cheng Su, Jinyu Huang, Suzhen Lin, Defu Li, Hong Cheng, Fu Wei, Songfeng Ou, Yunfeng Zou, Xiaobo Yang

**Affiliations:** 1 Department of Occupational Health and Environmental Health, School of Public Health, Guangxi Medical University, Shuangyong Road 22, Nanning, 530021 China; 2 Department of Neurology, The First Affiliated Hospital of Guangxi Medical University, Nanning, Guangxi China; 3 The First Clinical Medical College, Guangxi University of Science and Technology, Liuzhou, Guangxi China; 4 Department of Toxicology, School of Public Health, Guangxi Medical University, Nanning, Guangxi China; 5 Guangxi Colleges and Universities Key Laboratory of Prevention and Control of Highly Prevalent Diseases, Guangxi Medical University, Shuangyong Road 22, Nanning, 530021 China; 6 Center for Genomic and Personalized Medicine, Guangxi Medical University, Shuangyong Road 22, Nanning, 530021 China

**Keywords:** Manganese-exposed workers healthy cohort, Biobank, Follow-up

## Abstract

**Background:**

Long-term excess exposure to environmental manganese (Mn) can lead to multi-system damage, especially in occupational populations. Therefore, we established a manganese-exposed workers healthy cohort (MEWHC), focusing on the systemic health effects related to Mn exposure. Here, we aimed to describe the follow-up activity for the MEWHC study and establish a standardized biological sample bank for the scientific management of high-quality biospecimens and the attached data from 2011 to 2017.

**Methods:**

Baseline examinations for onsite workers were conducted, and the biobank for the MEWHC was first established in 2011; follow-up examinations occurred four times between July 2012 and November 2017. All questionnaires, clinical data and biological samples were routinely collected during each follow-up activity. Additional workers were recruited in 2016, which further enriched the resources of the biobank.

**Results:**

A total of 2359 onsite workers and 612 retired workers at a ferromanganese refinery were enrolled in the prospective cohort, and their biological samples were obtained in the preliminary baseline survey and the follow-up investigation, including 2971 blood and urine samples from the cohort. In addition, 1524 hair samples, 1404 nail (toe and finger nails) and 1226 fecal samples were also collected. All specimens were preserved in the biobank, and the data were scientifically managed using a computer system.

**Conclusions:**

The MEWHC study in China provides an effective way to obtain biological samples such as plasma, DNA, hair and urine for storage in a biobank for further study. The standardized management of various samples is crucial for accessing high-quality biospecimens.

**Electronic supplementary material:**

The online version of this article (10.1186/s12889-018-5880-0) contains supplementary material, which is available to authorized users.

## Background

The element manganese (Mn) is essential for normal physiological functions in the human body. However, excessive Mn exposure can be toxic to the human body, causing particular harm to the central nervous system [1, 2]. Humans are primarily exposed to Mn through their diet; however, another major route of exposure is via inhalation [3–5]. Overexposure may occur in occupational settings, especially from Mn mining dusts and welding fumes [6, 7]. Previous studies have shown that workers exposed to welding fumes are much more likely to experience extrapyramidal, Parkinsonian-type movement disorder than matched controls [8]. Furthermore, people residing near factories that utilize Mn in production are also threatened [9].

In recent years, prospective and retrospective methods have been applied to explore the effects of Mn exposure on various organs. However, the exact mechanism by which Mn exerts toxicity in humans has not yet been revealed. Thus, to explore Mn-induced toxicity in the human body, especially regarding the systemic health effects of Mn exposure, we established a prospective Mn-exposed worker healthy cohort (MEWHC), which began in 2011 in China [10]. To date, investigations of the subjects in the cohort have included numerous aspects related to the early health effects of Mn exposure, such as lifestyle habits and socio-economic status as well as environmental, occupational and genetic factors. Furthermore, we have also collected a range of biological samples, including blood, urine, hair, and nails, to explore early health effects, potential biomarkers of exposure, disease, susceptibility and diseases related to occupational Mn exposure.

Biobanks form a chain of operations that include informing subjects and obtaining proper consent (depending on local requirements), data acquisition, biospecimen collection, annotation, preservation, storage, quality control, cataloguing, managing of access, processing and distribution [11]. They have gradually begun to play an increasingly important role in studying the determinants of population health and shaping proper prevention programs and health policies. Therefore, it is necessary to establish a standardized biobank to preserve and manage specimens for ongoing research. Consequently, the MEWHC biobank will provide researchers with a reliable way to access high-quality biospecimens with clinical data for basic scientific research and validation studies. In addition, specimen resources, such as serum, plasma and human tissues, are limited and valuable, which requires us to manage and use these samples and their related data reasonably and effectively.

## Methods

### MEWHC study design and follow-up

**Fig. 1:**
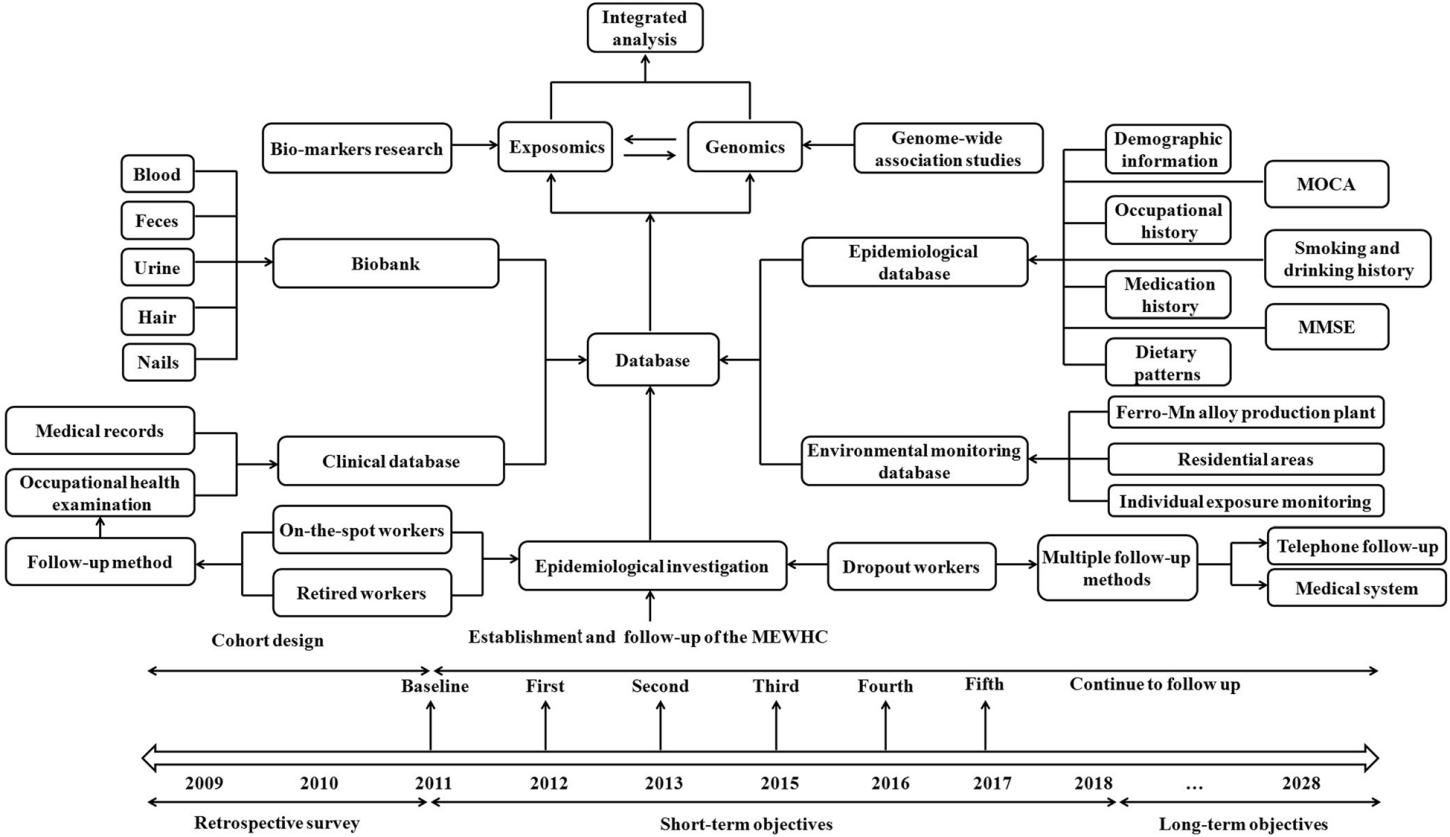
Study plan of the manganese-exposed workers healthy cohort (MEWHC). MoCA: The Montreal Cognitive Assessment; MMSE: Mini-mental State Examination

The prospective cohort study and the biobank for Mn-exposed workers began simultaneously in 2011. Health examinations of the participants are conducted annually. In our long-term follow-up survey, an increasing number of participants continue to be recruited into this heavy metal cohort, and the corresponding biobank and epidemiological data have been further enriched. The participants recruited into the cohort participate in annual follow-ups, and a comprehensive follow-up visit for all subjects is conducted every 3 years. As shown in Fig. 1, the biobank was established through the MEWHC study. The health examinations, questionnaires, files and environmental monitoring of the workers were conducted at baseline or via follow-up surveys. The collection of biological samples and related information includes a range of clinical, epidemiological and biological data, with integrated analysis by researchers.

### Standard protocol approvals, patient consent and investigator training

All study protocols were reviewed and approved by the Medical Ethics Committee of Guangxi Medical University. All participants provided written consent to participate prior to the start of the study. Workers exposed to Mn were recruited from one large ferro-Mn alloy production plant in Guangxi, China. All of the subjects had worked in the ferromanganese refinery for at least 1 year and lived in the local area.

All investigators were trained in how to obtain and process biological samples. These instructions covered the importance of specimens, sample information recording, sample processing, packaging, labeling, sample transportation and preservation. Focusing efforts on stringent quality control and quality assurance measures in establishing the biobank are important first steps [12]. The investigator must focus on not only collecting enough specimens but also ensuring that the collected specimens are of the necessary quality. Moreover, the executives of the biobank require a certain level of experimental skill and management ability to ensure the realization of the entire process, ranging from sample collection to detection data feedback.

## Sample collection

**Fig. 2:**
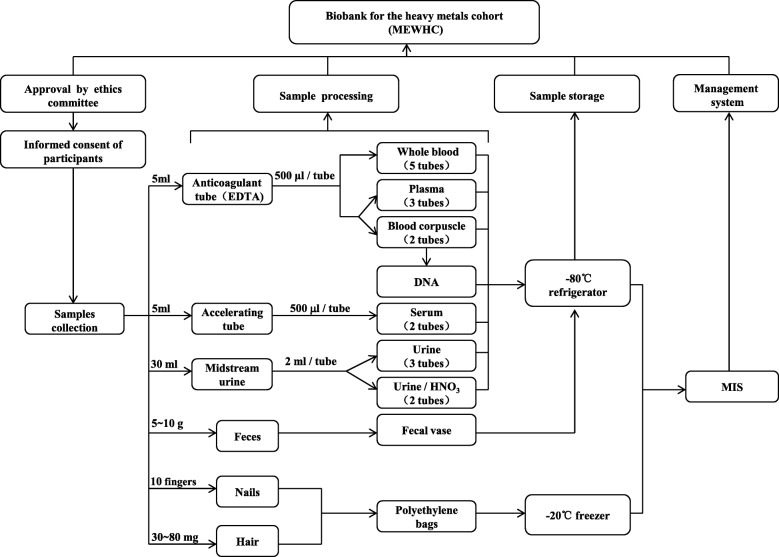
Flowchart of biological specimen procurement and preservation. MEWHC: Manganese-exposed workers healthy cohort; EDTA: Ethylenediaminetetraacetic Acid; MIS: Management Information System

All individuals from the MEWHC were informed about health examinations, questionnaire investigations and donating biological samples. The most important step in the workflow of biospecimen acquisition and preservation is that the researchers must strictly comply with the rules of operation. They must ensure that the timing of all operations is consistent with both the requirements of optimal collection and the preservation of biological products, meaning that the sample storage conditions were comparable to other large-scale biobanks. Detailed information on biological specimen collection is presented as a flowchart in Fig. 2.

### Collection of blood specimens and DNA extraction

Blood samples were collected from workers with different cumulative exposure indexes (CEIs) from the 8-h air Mn time-weighted average (TWA) in the occupational environment. At the first investigation and during the follow-up visits every 2 years, 5 mL of peripheral venous blood was collected in EDTA tubes. The samples were centrifuged using a high-speed benchtop centrifuge (Thermo Fisher, Biofuge Stratos) to separate plasma, serum and blood cells, and they were then stored at − 80 °C (Thermo Fisher, U.S.A). We extracted DNA from blood collected in EDTA tubes using Takara Universal Genomic DNA Extraction kits based on the manufacturer’s protocol (Takara, Japan).

### Collection of nail and hair specimens

A sufficient quantity of nail samples was available for metal assays. The nail clippings were collected from the study participants who had an adequate toenail length, including all 10 toenails and 10 fingernails. Before sample processing and testing, all nail specimens were placed in small polyethylene bags, stored in a clean container with desiccant and then placed in a − 20 °C freezer (KK25V61TI, 254 L, Haier) in the laboratory. The nail clippings were analyzed as previously described in Kile et al., in 2007 [13]: they were sonicated for 15 min in ~ 10 mL of 1% Triton X-100 solution, rinsed with distilled deionized water, dried at 60 °C in a drying oven for 24 h, weighed, digested in nitric acid and analyzed using inductively coupled plasma mass spectrometry (ICP-MS) (Nex10N350x, Agilent, USA).

Hair specimens were taken on the same day as other samples. Approximately 80 mg of hair was taken from the nape of the head as close as possible to the scalp with stainless-steel scissors and stored in polyethylene bags. Hair samples were stored in the same way as nail samples before they were sent for trace element analysis. According to the modified method developed by the International Atomic Energy Agency [14], the hair specimen was cut into pieces, washed in deionized water, rinsed with acetone and then dried in an oven at 50 ± 10 °C. After drying, the hair samples were digested with nitric acid and then diluted with deionized water containing 1% nitric acid. Finally, the hair samples were analyzed by inductively coupled plasma-mass spectrometry (ICP-MS) (Nex10N350x, Agilent, USA).

### Collection of urine and fecal specimens

A midstream urine sample (> 30 mL) was collected in a plastic container, acidified to 1% (*v*/v) HNO_3_, divided into aliquots and subsequently stored in polyethylene tubes at − 20 °C until shipment to Guangxi Medical University, where samples were stored frozen (− 80 °C, Thermo Fisher, USA) prior to further determination of Mn and trace elements levels.

We started the collection of fecal samples in 2016. Accounting for the feasibility of on-site fecal specimen collection, including time limitations and the participants’ compliance, we decided to collect the fecal specimens from retired ferroalloy plant workers. Following enrollment, participants were invited to provide a fresh fecal specimen. Immediately following collection, the samples were placed in a sterile feces tube containing no solution, and then the study investigators sealed the feces tubes and froze each specimen (− 80 °C, Thermo Fisher, USA). The average time from sample collection to storage was less than 2 h.

### Data collection

Data collection included questionnaires, information on environmental exposure and clinical data.

The questionnaire collected information on demographics, socio-economic status, smoking history, alcohol consumption, medication history and occupational history. Smoking habits were classified as smoker (smoking at least 1 cigarette daily for more than 3 months) or non-smoker (never smoked or a former smoking habit with cessation for more than 3 months). Drinking habits were classified as current drinker (drinking at least once each week for more than 3 months) or nondrinker (never drank or formerly drank) [15]. In addition, 2 neurological function tests were conducted to assess and screen for the effects of Mn exposure on the nervous system using the following methods: the Montreal Cognitive Assessment (MoCA), a neurocognitive function test that rapidly screens for mild cognitive impairment with high sensitivity and specificity [16–19]; and the mini-mental state examination (*MMSE*), a scale for screening dementia that can comprehensively, accurately and rapidly reflect the intelligence status and cognitive impairment degree of the subjects [17, 19–21]).

The data on environmental exposure were divided into approximately 2 parts, including harmful occupational factors in the factory and the levels of Mn in the air in the residential area. We recorded the production processes and conditions of potential Mn pollution in this plant and detected the individual levels of Mn using individual samplers. The method used has been described in detail in previous studies [22]. We then calculated the cumulative exposure index (CEI) from the 8-h air Mn TWA in each department of the factory and multiplied it by the Mn exposure-years for each worker. In particular, we performed more comprehensive air monitoring through personal samplers according to the participants’ occupations in 2017. This method enabled the exploration of accurate exposure levels among the participants in different occupations. More detailed information on personal samplers according to their occupations has been described in (Additional file 1: Table S1). Information about the jobs and tasks of the subjects has been described in detail in previous studies [10].

Clinical data were obtained from medical records, which were matched to the stored specimens during every epidemiological investigation. Baseline and follow-up surveys were conducted face-to face or via telephone interviews. Then, all of the available data were recorded and stored in information databases or the biobank.

### Biobank management system

A biobank management system mainly includes a hardware facility system, a monitoring system (temperature monitoring) and an information management system. The hardware facility system was equipped with an electronic system and refrigeration and included a 4 °C refrigerator, a − 20 °C freezer, a − 80 °C low-temperature freezer and a − 152 °C liquid nitrogen tank. The management system, as mentioned above, was used to manage all biological samples and related data, especially the detailed records of the participants’ exposure history in each follow-up activity, which was controlled by professionally trained staff. Secure data storage and high-quality digital preservation and annotation of information related to the biosamples are indispensable. One staff member was trained to supervise collecting and inputting all clinical information.

Each subject has a unique identifier (Barcode printer, B-2404, TSC, China), which can facilitate linking different databases and helps researchers to conveniently manage biospecimens. Furthermore, there are strict rules for the use of samples, which must be recorded in detail for any experiment on the samples and under the supervision of the bank manager. In that case, biobank users must be able to fully track samples in interoperable electronic systems that pay particular attention to the quality and security of the data. More information can be found in Additional file 2.

### Statistical analysis

A Chi-squared test was used to perform statistical analyses of the frequency data. The significance level for all statistical tests was 0.05. All statistical analyses were performed using the software package SPSS (Version 21.0).

## Results

### Demographic characteristics of the cohort

As shown in Table 1, the basic characteristics of 2971 workers included retired workers (*n* = 612) and onsite workers (*n* = 2359). The retired worker group consisted of 231 men (37.75%) and 381 women (62.25%), and the onsite worker group consisted of 1453 men (61.59%) and 906 women (38.41%). The average age of the retired workers was 69.7 years, and that of the onsite workers was 40.0 years. Of the retired workers, 63 (10.29%) were current smokers, and 132 (21.57%) were current drinkers. Of the onsite workers, 877 (37.18%) were current smokers, and 1071 (45.40%) were current drinkers, and the current passive smoking rates were 46.92 and 85.67%, respectively. Based on the biobank for the heavy metal cohort, except for the laboratory tests, which included routine blood tests, routine urine tests and liver function tests, we detected levels of Mn and Fe in blood, urine or hair using atomic absorption spectrometry. In this case, we combined the environmental exposures provided at the level of each cohort member with other individual-level data and biobank samples for further epidemiological analysis.

**Table 1:** Demographic data of the study population

Variables	Onsite workers		Retired workers		*P*
	Number	Percent	Number	Percent	
	(*n* = 2359)		(*n* = 612)		
Sex					
Male	1453	61.59	231	37.75	0.00
Female	906	38.41	381	62.25	
Age, years					
< 30	249	10.56	0	0.00	0.00
30–40	818	34.68	0	0.00	
40–50	1100	46.63	4	0.65	
50–60	185	7.84	105	17.16	
60–70	7	0.30	159	25.98	
70–80	0	0.00	245	40.03	
≥ 80	0	0.00	99	16.18	
Race/ethnicity					
Han Chinese	1148	48.66	401	65.52	0.00
Zhuang minority	1109	47.01	189	30.88	
Other ethnic groups	92	3.90	21	3.43	
Missing	10	0.42	1	0.16	
Marital status					
Single	293	12.42	11	1.80	0.00
Married	1989	84.32	451	73.69	
Widowed or divorced	73	3.09	149	24.35	
Missing	4	0.17	1	0.16	
Education					
Middle school or lower	948	40.19	470	76.80	0.00
High school	1050	44.51	111	18.14	
University/college or higher	358	15.18	29	4.74	
Missing	3	0.13	2	0.33	
Seniority, years					
< 10	775	32.85	8	1.31	0.00
10–20	750	31.79	90	14.71	
20–30	693	29.38	260	42.48	
≥ 30	138	5.85	253	41.34	
Missing	3	0.13	1	0.16	
Smoking status					
Current smoker	877	37.18	63	10.29	0.00
Former smoker	160	6.78	81	13.24	
Never smoker	1317	55.83	466	76.14	
Missing	5	0.21	2	0.33	
Drinking status					
Current drinker	1071	45.40	132	21.57	0.00
Former drinker	386	16.36	102	16.67	
Never drinker	896	37.98	374	61.11	
Missing	6	0.25	4	0.65	
BMI, kg/m^2^					0.00
< 18.5	88	3.73	9	1.47	
18.5–24	1445	61.25	232	37.91	
24–28	670	28.40	251	41.01	
≥ 28	136	5.77	94	15.36	
Missing	20	0.85	26	4.25	
Height, cm (mean ± SD)	163.18 ± 7.56		155.29 ± 7.78		
Weight, kg (mean ± SD)	60.46 ± 9.95		57.99 ± 9.68		

*BMI* body mass index. *SD* standard deviation

### Establishment of the biobank

During the 6-year active period of this prospective longitudinal study, 2359 onsite workers and 612 retired workers were enrolled in the heavy metal cohort for occupational health examination, and series of different types of biological samples were obtained, including blood and urine samples of each participants, 1226 fecal samples, 1404 nail samples and 1524 hair samples. The results of the sample types and all of the numbers of biosamples collected from 2011 to 2017 are shown in (Additional file 3:Table S2). In addition, we monitored the occupational hazardous factors in the working environment of the research objectives, especially the level of Mn dust in the air.

### Findings based on the MEWHC study

Currently, the data show that working with ferroalloy is related to reduced lung ventilation function. Furthermore, the MEWHC study also showed that Mn exposure is related to decreased lung ventilation function in male smelter workers; individual smoking habits and Mn exposure have a synergistic effect on decreased lung function [23]. Liver function was compared among the different Mn-exposed groups in 2013, and we found that occupational Mn exposure can cause a dose-dependent increase in liver enzyme levels and can interact with alcohol consumption to aggravate liver damage [10]. Regarding the biomarkers of exposure, disease and susceptibility, the levels of plasma brain-derived neurotrophic factor (BDNF) and dopamine were determined using sandwich ELISA kits (ChemiKine, USA), and the biomarkers of susceptibility were assessed using genome-wide association studies (GWAS) [10]. The results showed that a decrease in plasma BDNF levels and cognitive impairment may be caused by occupational Mn exposure.

## Discussion

Banking biosamples from population-based cohorts holds significant research potential today, and the MEWHC biobank has been the core of the heavy metal cohort. The present biobank also offers an open research platform to provide an integral resource for biological samples, including blood, plasma, DNA and human tissues, which can be used for the discovery, validation and implementation of biomarkers or potential new biomarkers of exposure, disease and susceptibility.

Thus far, we have investigated a series of biomarkers based on the MEWHC biobank, especially biomarkers for exposure and susceptibility. Our results suggest that Mn in blood cells might serve as a biomarker of Mn exposure [24]. Larger validation studies using the rich biological biosamples of the MEWHC biobank are needed to determine the utility of this biomarker. Furthermore, our results show that Mn exposure may be associated with cognition impairment in this large cross-sectional study. There is evidence to suggest that higher serum BDNF levels are associated with better neuropsychological function [25]. Thus, the detection of biomarker BDNF was performed immediately, and animal and molecular biology experiments were also conducted to investigate the role of BDNF. A negative dose–response relationship between plasma BDNF and Mn exposure levels was observed. Therefore, plasma BDNF levels can be a potential marker of cognitive impairment induced by Mn exposure [22].

Since the establishment of the biobank, its researchers have been committed to the standardization of biospecimen management. The entire sample management process, including collection, transport, processing and storage, will be strengthened, especially in the following three aspects: preventing samples from being polluted by various biomolecules throughout the management process, from collection from the Mn-exposed workers to storage in the biobank; controlling time and temperature in sample transportation, both of which affect biospecimen quality; and establishing standard operating procedures for the centrifugation of blood, DNA extraction and anticorrosion of urine, among others.

We also focused on how to maximize the research usability of the biospecimens since the number of biospecimens is limited [26]. In the past, samples used for Mn analysis to explore associations between Mn exposure and concentrations in biosamples were examined using traditional atomic absorption spectrometry; however, we can now detect the concentrations of 22 metals using inductively coupled plasma mass spectrometry (ICP-MS). Experimenters should be required to optimize their use of biosamples to maximize opportunities for further research based on these biosamples [27]. At present, new technologies are becoming more refined so that even very small amounts of sample can become rich sources of discovery; in particular, the biospecimens from patients diagnosed with Mn poisoning, which are few and precious, will be a powerful resource in the Mn-exposed cohort to support mechanism research. Therefore, the dropout rate of the workers is still a limitation of the current research—the dropout rate of the MEWHC was 23.23% (548/2359). Due to the dynamic characteristics of the MEWHC, one of the largest challenges for specimen collection is reducing the dropout rate. In recent years, some workers have chosen to leave or transfer to civilian work for various reasons.

To address these challenges, we have primarily adopted 3 types of follow-up methods according to the reasons for the dropout. In the future, we will continue to organize and conduct occupational health examinations for onsite or retired workers. Additionally, we also telephone the subjects who are leaving the area or transferring to civilian work to obtain information, and even track outcomes, through the medical system.

In addition, the MEWHC biobank has made a significant contribution to the present research findings and provides rich sample resources for further research. It should be open to external researchers as a platform that allows scholars to search for and identify biospecimens in order to develop and validate new value-based biomarkers. The biobank aims to facilitate data sharing and pooling across multiple population-based biobanks of heavy metal cohorts to stimulate cooperation among investigators and to maximize the utility of biospecimens in research. Furthermore, the MEWHC biobank also provides an open channel to share the research findings generated using human biospecimens; therefore, it can contribute to many research projects.

## Conclusions

The biosamples stored in the MEWHC biobank, such as plasma, DNA, hair and urine, will make important contributions toward the discovery and validation of biomarkers. Thus far, the MEWHC biobank has played important roles in epidemiology studies and in exploring the genetic susceptibility of Mn exposure. Further analyses of information from GWAS, combined with the results from molecular toxicology analyses, are crucial to facilitating our future studies. Therefore, the longitudinal MEWHC will be continued, and the standardization of the biobank management system and expansion of the specimen database are necessary.

## Additional files

Additional file 1: **Table S1.** Air monitoring of different workshops and jobs in the ferro-Mn alloy production plant in 2017. (PDF 107 kb)

Additional file 2: Supplementary information for the biobank of the MEWHC. (PDF 398 kb)

Additional file 3: **Table S2.** Summary of the number of samples deposited in the biobank obtained from the heavy-metal cohort (MEWHC) initiated in 2011. (PDF 89 kb)

## Abbreviations

BDNF: Brain-derived Neurotrophic Factor
BMI: Body Mass Index
CEI: The Cumulative Exposure Index
EDTA: Ethylenediaminetetraacetic Acid
GWAS: Genome-wide Association Studies
ICP-MS: Inductively Coupled Plasma Mass Spectrometry
MEWHC: Manganese-exposed Workers Healthy Cohort
MIS: Management Information System
MMSE: Mini-mental State Examination
MoCA: The Montreal Cognitive Assessment
TWA: Time-weighted Average
