# Supplementary material for: Follow-up of the manganese-exposed workers healthy cohort (MEWHC) and biobank management from 2011 to 2017 in China
Source: BMC Public Health. 2018 Aug 1;18:944. doi: 10.1186/s12889-018-5880-0 (PMC6090756; doi:10.1186/s12889-018-5880-0)
Supplement: Supplementary file 1 — Table S1. Air monitoring of different workshops and jobs in the ferro-Mn alloy production plant in 2017. (PDF 107 kb) [file 12889_2018_5880_MOESM1_ESM.pdf]

Supplement Table 1 Air monitoring of different workshops and jobs in the ferro-Mn alloy production plant in 2017.

Branch	Workshop	Job title	Number
Smelting branch	Smelting workshop No.1	Material worker	3
		Furnace face worker	3
		Electrician	3
		Scaleman	3
		Craneman of casting	3
		Furnaceman	3
		Dedusting workers	3
	Smelting workshop No.2	Material workers	3
		Furnace face worker	3
		Electrician	3
		Scaleman	3
		Craneman of casting	3
		Furnaceman	3
		Dedusting workers	3
	Smelting workshop No.3	Craneman of material	3
		Material workers	3
		Furnace face worker	3
		Electrician	3
		Scaleman	3
		Craneman of casting	3
		Furnaceman	3
		Dedusting workers	3
	Smelting workshop No.4	Furnace face worker	3
		Electrician	3
		Scaleman	3
		Craneman of casting	3
		Furnaceman	3
		Dedusting workers	3
	The maintenance workshop	Welder	3
		Fitter	3
Logistics branch	The maintenance workshop	Welder	3
		Fitter	3

	Raw material workshop	Sieving workers	3
		Plugman	3
	Testing laboratory	Chemical analyst	3
		Sampleman (stokehole)	3
		Sampleman (material)	3
	Finished products workshop	Human crushing worker	8
		Loading workmen	2
Others	Outside the office	Administration staff	3
	Inside the office	Administration staff	3
	Staff Dining Hall	Cook	3
	Switching room	Blank control group	5
Total			135
