# Supplementary material for: Follow-up of the manganese-exposed workers healthy cohort (MEWHC) and biobank management from 2011 to 2017 in China
Source: BMC Public Health. 2018 Aug 1;18:944. doi: 10.1186/s12889-018-5880-0 (PMC6090756; doi:10.1186/s12889-018-5880-0)
Supplement: Supplementary file 2 — Supplementary information for the biobank of the MEWHC. (PDF 398 kb) [file 12889_2018_5880_MOESM2_ESM.pdf]

Supplementary information for the biobank of the MEWHC

The biobank of the MEWHC was established in 2011, and it provids researchers a reliable way to access to high-quality biospecimen with clinical data for basic scientific research and validation study. Therefore, a standardized bio-bank management system will be necessary to preserve and manage the precious specimens for the ongoing researches. The management system of the biobank mainly included hardware facility system, monitoring system (temperature monitoring) and information management system.

The hardware facility system was equipped with an electronic system and refrigerations, including 4℃ refrigerator, -20℃ freezer, -80℃ low-temperature freezer, and -152 ℃ liquid nitrogen tank.

The monitoring system (temperature monitoring) can be used as an effective tool for the power failures or the temperature exceeding the threshold in the refrigerator. Once the power failures or the temperature exceeds the threshold, the refrigerator will automatically alarm and send a corresponding prompt information to the cellular phone of manager for checking and repairing. Besides, "Backup system" (CO_2 / LN_2 radiator cooler) will be applied to the biobank for the problem of power failure. In case of the power failures, the refrigerator with the liquid carbon dioxide (-37°C) will keep at low temperature (-65 ~ -70°C) for 10 hours.

The information management system was used to manage all biological samples and related data, especially the detailed record of the exposure history of the participants in each follow-up activity. The information management system was

controlled by staffs who received professional training. The training instructions consisted of the importance of specimens, sample information record, sample processing, packaging, labeling, sample transportation and preservation.

Each subject has a unique identifier (Barcode printer, B-2404, TSC, China), which can facilitate the linkage of different databases and helps to manage biospecimens conveniently for researchers. The different type of samples collected from workers also have a unique barcode. The detailed information of each sample in the information management system includes the following aspects: a) demographic data of the subjects (unique identifie, sex, age, etc.) and b) information of the sample (type, volume, acquisition time, etc.).

The collection procrdure of samples was described in detail in the manuscript, and the sample processing was showed in the Figure 2. Then the cryogenic vials are stored in the support rack according to the table of pre-assigned position information.

Finally, The following pictures were attached to show the storage process of samples.

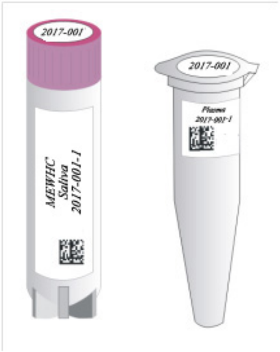

a. The tubes with barcode.

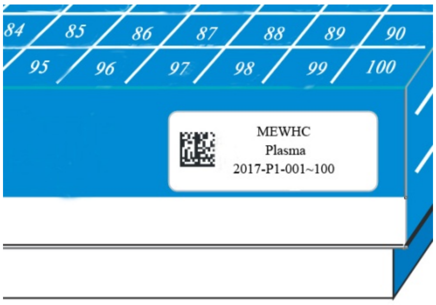

b. Frozen box with barcode and location code.

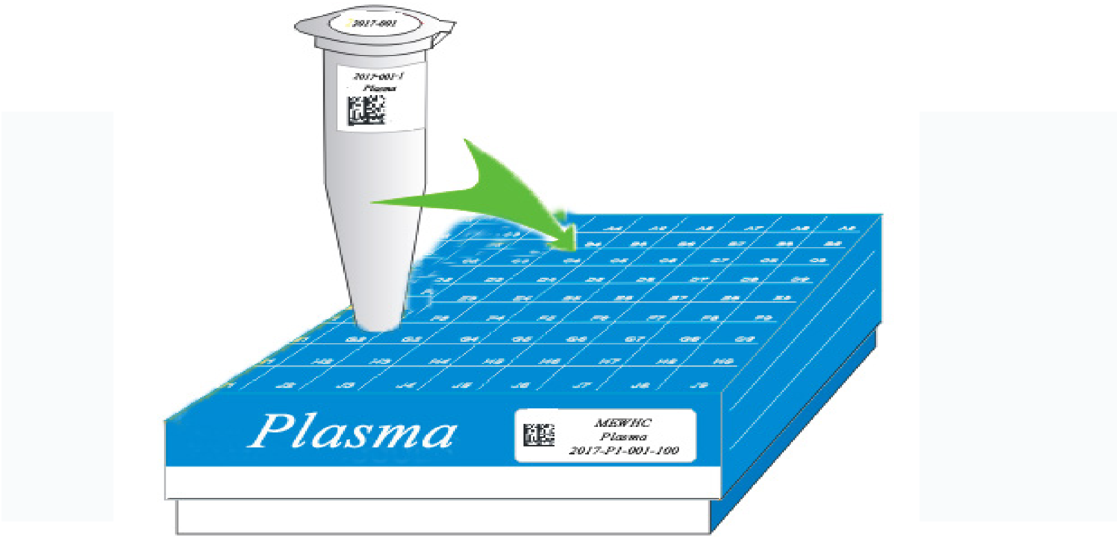

c. Storing samples by category and labeling.

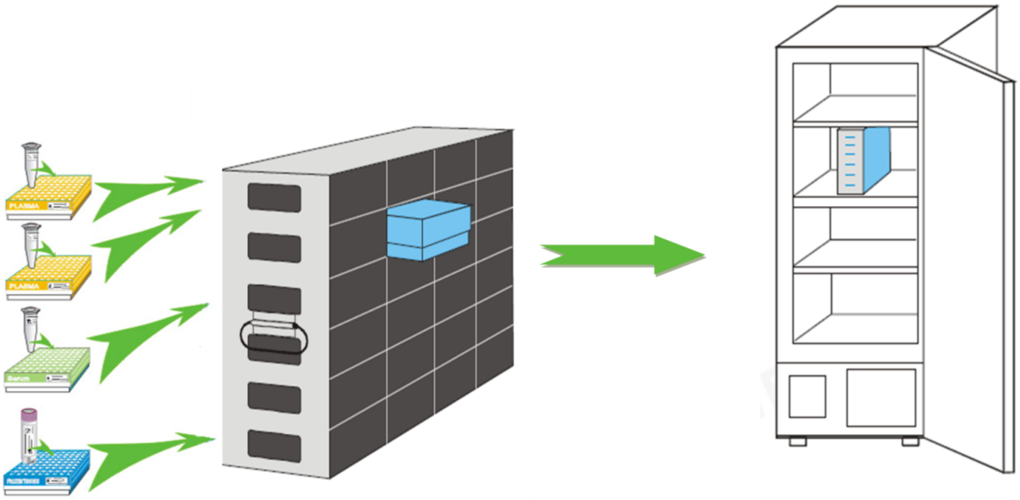

d. Storing samples in the refrigerator.
