# Supplementary material for: Follow-up of the manganese-exposed workers healthy cohort (MEWHC) and biobank management from 2011 to 2017 in China
Source: BMC Public Health. 2018 Aug 1;18:944. doi: 10.1186/s12889-018-5880-0 (PMC6090756; doi:10.1186/s12889-018-5880-0)
Supplement: Supplementary file 3 — Table S2. Summary of the number of samples deposited in the biobank obtained from the heavy-metal cohort (MEWHC) initiated in 2011. (PDF 89 kb) [file 12889_2018_5880_MOESM3_ESM.pdf]

Supplement Table 2 Summarizes all the number of samples deposited in the biobank obtained from the heavy metal cohorts (MEWHC) initiated in 2011.

Sample type	On-the-spot workers (n=(2359)		Retired workers (n=(612)		Total
	Number	Percent	Number	Percent	Number
Blood / DNA	2359	100	612	100	2971
Urine samples	2359	100	612	100	2971
Fecal samples	719	30.5	507	82.8	1226
Hair samples	931	39.5	593	96.9	1524
Nail samples	811	34.4	593	96.9	1404
